# Prescription of benzodiazepines and antidepressants among Sami and non-Sami — How childhood violence shapes prescription patterns: the SAMINOR 2 questionnaire survey and the Norwegian prescription database

**DOI:** 10.1186/s12889-024-20570-1

**Published:** 2024-11-07

**Authors:** Astrid M. A. Eriksen, Marita Melhus, Ann-Ragnhild Broderstad, Janet Smylie

**Affiliations:** 1 Centre for Sami Health Research, Department of Community Medicine, UiT The Arctic University of Norway, Tromsø, Norway; 2 Dalla Lana School of Public Health and Department of Family and Community Medicine, Faculty of Medicine, University of Toronto, Toronto, ON Canada

**Keywords:** Childhood violence, Antidepressants, Benzodiazepines, Ethnicity, Sami, SAMINOR, Norwegian prescription database

## Abstract

**Background:**

Medication for mental health problems represents a significant proportion of overall medication use and the prescription of psychotropic medicine has increased in many western countries over the last decades. Childhood violence (CV) is strongly associated with mental health problems, which in turn may increase the likelihood of being prescribed psychotropic medication. However, the association between CV and prescription of benzodiazepines (BDZ) and antidepressants is rarely described, and no such study has been performed among the Indigenous Sami people.

**Methods:**

Data from the SAMINOR 2 Questionnaire Survey (2012) was linked to the Norwegian Prescription Database. Information on filled prescriptions for BDZ and antidepressants in 2004–2019 was collected for 11,296 persons (55.8% women, 22.6% Sami). Gender-stratified chi-square tests and two-sample *t*-tests were used to test for differences between groups. Logistic regression was applied to investigate the association between CV and filled prescriptions for BDZ and antidepressants.

**Results:**

During the 16-year study period, 16.7% of all women filled at least one prescription for BDZ. The figures were significantly lower among Sami women (14.1%) compared to non-Sami women (17.4%) (*p* = .003). Among all women, 23.6% filled at least one prescription for antidepressants, with no difference between ethnic groups. Filled prescriptions among men were 10.0% and 14.2%, respectively, with no difference between ethnic groups. During each year, and in total, a significantly higher proportion of women exposed to CV received at least one prescription for BDZ and antidepressants, respectively, compared to women not exposed to CV, with no differences between ethnic groups. Among men, the pattern was similar.

**Conclusion:**

A lower proportion of Sami women filled prescriptions for BDZ than non-Sami women. Those who reported exposure to CV filled prescriptions for BDZ and antidepressants more often than those who did not report CV. There were no overall differences between Sami and non-Sami; the dispensing rates of antidepressants and BDZ were similar for Sami and non-Sami, and the effects of CV on the dispensing of antidepressants and BDZ were also similar. This study highlights the importance of preventing CV, and of identifying a history of CV when treating adults with mental health problems.

**Supplementary Information:**

The online version contains supplementary material available at 10.1186/s12889-024-20570-1.

## Background

The long-term influence of childhood violence on prescription of psychotropic medications in adults is rarely described. Childhood violence encompasses various forms of maltreatment, including physical, emotional, and sexual abuse, as well as neglect [1]. The World Health Organisation (WHO) highlights numerous health risks linked to childhood violence, particularly concerning mental health [1, 2]. Conditions such as depression, anxiety, post-traumatic stress disorder, and substance abuse are notably more prevalent among those who have experienced violence and abuse during childhood [3–6]. Exposure to childhood violence (CV) is recognised as a public health problem due to the impact of mental health issues that hinder individuals’ ability to realize their potential, cope with everyday stresses, work effectively, and engage in their communities [1, 2]. However, the cause of mental health problems is multifactorial and can be attributed to genetic factors, various psychological and environmental stressors, including trauma, and a variety of other diseases and disease processes. The use of benzodiazepines (BDZ) and antidepressants in treatment for mental health problems, such as anxiety and depression, is well established [7].

Medication for mental health problems represents a significant proportion of overall medication use and the prescription of psychotropic medicine has increased in many western countries over the last decades [8, 9]. In Norway, use of BDZ increased in the years 2004–2008/2010, after which it decreased towards 2020 [10]. Use of antidepressants has been relatively stable, with a slight increase in 2020 and 2021 [11]. In 2021, 4.5% of the population in Norway had at least one filled prescription for BDZ and 7% had at least one filled prescription for antidepressants [11]. BDZ carry the risk of adverse side effects such as anxiety, mood swings and sleep disturbance, cognitive impairment and falls [13–14]; and antidepressants carry the risk of emotional numbness, sexual dysfunction, nausea and sleep disturbance [15, 16].

Studies have shown disparities across ethnic groups in terms of filled prescriptions for psychotropic medication [17–21]. One study among the US adult population found that non-Hispanic, Black and Mexican-American males and females had a lower prevalence of psychotropic medication use compared to non-Hispanic whites. Cook et al. found that Blacks and Latinos were less likely to fill prescriptions for any psychotropic medication [21]. A study among the elderly in New Zealand found a lower prevalence of antipsychotic and antidepressant medication use among Māori compared to non-Māori [20]. Differences in culture between physicians and minority patients can hinder communication, potentially affecting the prescription of medications.

The Sami is an European indigenous people, and have their own language and culture [22]. Traditionally they have mainly populated Northern Europe, northernmost parts of Norway, Sweden and Finland, and the Kola Peninsula of the Russian Federation. In Norway, Sami communities are also found further south and many Sami also live in cities [23]. The Sami have been subjected to assimilation and discrimination for over hundred years [22, 24]. State policy to assimilate the Sami started around 1860 and had devastating consequences including restriction on speaking Sami language in schools and limited opportunities for developing and preserving Sami culture. The main goal of this state policy was to wipe out Sami language and culture. The Truth and Reconciliation report from 2023 thoroughly documented this hash assimilation policy, the widespread discrimination and unjust treatment of the Sami people in Norway [22]. Previous research has shown that Sami to a greater extent than non-Sami reported having been subject to CV, and a higher prevalence of mental health problems [25] Further, an association was found between CV and mental health problems in adulthood [26]. This has been linked to the historical trauma of the Sami as well as other indigenous populations globally [27–29].

There is limited knowledge regarding the use of psychotropic medication among the Sami people, and to our knowledge, there are no studies focusing on the association between CV and the use of psychotropic medication as adults in a population of Sami and non-Sami. Since a higher prevalence of mental health problems is found among Sami than non-Sami, we hypothesised a higher prevalence of filled prescriptions for BDZ and antidepressants among Sami than non-Sami.

Considering the literature addressing the association between CV and mental health problems, we hypothesised a higher prevalence of BDZ and antidepressants among those exposed to CV than those not exposed to CV. We also hypothesised a similar prevalence of prescriptions among Sami and non-Sami women exposed to CV, but possibly a different pattern among Sami men than non-Sami men, based on previous research showing that there are no ethnic differences among women with respect to disclosure of exposure to violence to professionals, but Sami men disclose less than non-Sami men [29]. Traumatic events like exposure to childhood violence can lead to unresolved trauma and mental health problems. There is some evidence that early disclosure of childhood abuse can protect from future mental health problems. Among those who have experienced childhood trauma, mental health problems may be more severe in situations where the trauma has not been disclosed [30]. In Sami child-rearing, particularly Sami men are taught to cultivate strength and to endure pain [31]. This might imply that Sami men are less likely to disclose abuse and/or seek help from professionals and hence are prescribed less psychotropic medication than non-Sami men. Conversely, Sami men may have more pronounced adult symptoms as a result of non-disclosure, leading to a higher likelihood of being prescribed psychotropic medication.

This exploratory study aimed to: (1) Estimate the prevalence of filled prescriptions for BDZ and antidepressants among Sami and non-Sami men and women; and (2) Explore the effect of reported exposure to CV on prescription rates for these medications across these subpopulations.

## Methods

In this cohort study, we used baseline data from the SAMINOR 2 Questionnaire Survey [32] and followed 11,063 persons for 16 years by linking their data to the Norwegian Prescription Database (NorPD) and national register data from Statistics Norway (SSB).

### Data sources

The SAMINOR 2 Questionnaire Survey (hereafter referred to as SAMINOR 2) was part of the second wave of the Population-based Study on Health and Living Conditions in Regions with Sami and Norwegian Populations (the SAMINOR Study). The survey was conducted by the Centre for Sami Health Research in the period January-October 2012. Everyone aged 18–69 years living in 25 pre-selected municipalities was invited (except for six of the municipalities, where only inhabitants of some districts of the municipality were included). All the selected municipalities are core areas of Sami settlement in Norway. Out of 43,245 invitees, 11,600 participated (response rate 27%). Details of the survey is previously described [32] and an English translation of the questionnaire is available on www.saminor.no.

The NorPD contains a complete list of all prescriptions dispensed by Norwegian pharmacies to non-institutionalized individuals. Drugs are classified by the Anatomical Therapeutic Chemical (ATC) system.

Statistics Norway (SSB) provides demographic data, including information about deaths. In this study, year of death was collected for the period from 1 January 2004 until 31 December 2019.

### Study period

The study period was defined as being from 1 January 2004 until 31 December 2019.

### Study population

Out of 11,600 participants, 304 were excluded due to missing information on key variables (violence and ethnicity), leaving 11,296 persons eligible for the study. Of these, 6,303 (55.8%) were women and 4,993 (44.2%) were men. For each year of follow-up, respondents who died were excluded from the sample, and this reduces the sample size in the final study sample in 2019.

### Variables from SAMINOR 2

Variables from the SAMINOR 2 Survey has previously been described in other articles [26, 33, 34].

#### Childhood violence

Three variables assessed exposure to emotional, physical and sexual violence during childhood, using questions from the Norvold Abuse Questionnaire (NorAQ).

Participants who answered “Yes, as a child” to at least one of the questions “Has anyone ever systematically and over a long period tried to subdue, degrade or humiliate you?”, “Have you experienced physical attacks/abuse?” and “Have you been sexually abused?” were classified as exposed to CV, with the remaining respondents classified as non-exposed.

#### Ethnicity

Sami ethnicity was classified if the respondents considered themselves Sami or stated a Sami ethnic background, and in addition reported that they themselves, or a parent or a grandparent, used Sami as their home language. The remaining respondents were classified as non-Sami. The majority of respondents in the non-Sami population define themselves as Norwegians (92.7%), the remaining 7.3% consist mainly of respondents from Nordic/northern European countries. This grouping resulted in 2,558 Sami (22.6%) and 8,738 non-Sami (77.4%).

#### Level of education

Level of education was classified into four groups according to the total number of years of completed education: 0–9 years (primary school), 10–12 years (secondary school), 13–15 years (college/university, 1–3 years), and 16 years and more (college/university, four years or more).

#### Mental health problems

Mental health problems were measured by the 10-item version of the Hopkins Symptom Checklist (HSCL-10) with a cut-off of ≥ 1.85. This version primarily measures symptoms of anxiety and depression during the previous four weeks. Each item was rated on a four-point scale, from 1 “Not affected” to 4 “Severely affected”. In accordance with Strand et al. [25], missing values were replaced with the sample mean of the specific item before the total score was calculated as the mean of the ten items.

### Age and gender and county of residence

Year of birth, gender and county of residence were retrieved from the National Population Registry. Age was set to attained age at 01.01.2012.

### Information on dispensing of BDZ and antidepressants from NorPD

The two outcomes in this study were the dispensing from Norwegian pharmacies of BDZ derivates (ATC codes N05BA) and antidepressants (ATC codes NO6A), respectively. For each calendar year of the follow-up period, a participant was categorised as a user of BDZ or antidepressants, respectively, if they received at least one prescription for the specific drug during the year. In addition, we calculated whether they received at least one prescription during the 16-year period.

### Analytic strategy and statistical analyses

All main analyses are stratified by gender. Descriptive statistics are presented for Sami and non-Sami. Ethnic differences in mean age are tested using two-sample t-tests. All other variables are categorical and presented as numbers and percentages. Pearson’s chi-square test was used to test for differences between groups. The number and percentage of BDZ and antidepressant users are presented for Sami and non-Sami for each year and for all 16 years combined, as well as for those reporting vs. those not reporting childhood violence. Separate logistic regression analyses were conducted, with BDZ and antidepressant use, respectively, as the dependent variable. Exposure to CV was used as the main predictor, with non-exposed as a reference, and we adjusted for age and educational level. Possible interaction between CV and ethnicity was also investigated.

All computations and statistical analyses were performed using IBM SPSS Statistics Version 28.0. The level of significance was set to 5%.

### Ethics

The SAMINOR 2 Questionnaire Survey was approved by the Norwegian Data Protection Authority (Datatilsynet). Written informed consent was obtained by all participants by returning the questionnaire. This study was approved by the Regional Committee for Medical and Health Research Ethics (REK-Sør-Øst, number 23,394), the SAMINOR Project Board, UiT the Arctic University of Norway, the Norwegian Agency for Shared Services in Education and Research, Statistics Norway (SSB) and the Norwegian Prescription Database (NorPD).

## Results

Sample characteristics are presented in Table 1 (women) and Table 2 (men). Sami women were significantly younger (mean age 45.7) than non-Sami women (mean age 46.9) (*p* = .002), while there was no difference in age between Sami and non-Sami men (mean age 49.7). Sami women reported a higher educational level than non-Sami women (*p* < .001), while there was no difference in educational level among men (*p* = .184) (Tables 1 and 2). A higher proportion of Sami women (15.9%) than non-Sami women (12.6%) (*p* = .002) reported mental health problems. The same pattern was observed among men; Sami (10.7%) vs. non-Sami 8.0%) (*p* = .005). A higher proportion of Sami reported CV than non-Sami; Sami women (31.4%) vs. non-Sami women (20.8%) (p = < 0.001), and Sami men (28.0%) vs. non-Sami men (15.6%) (*p* < .001).

**Table 1:** Background characteristics among all women and by ethnicity. The SAMINOR 2 Questionnaire Survey and the Norwegian prescription database 2004–2019

	All women % *n* = 6303	Sami % *n* = 1454	Non-Sami % *n* = 4849	*P*
**Age (years)**, **mean (SD)**	46.6 (13.7)	45.7 (14.0)	46.9 (13.7)	0.002
**Educational level**, **% (n)**				< 0.001
Primary school	12.4 (775)	12.3 (177)	12.5 (598)	
Secondary school	24.2 (1505)	19.8 (284)	25.5 (1221)	
College/university 1–3 years	26.5 (1651)	25.6 (368)	26.8 (1283)	
College/university ≥ 4 years	36.9 (2300)	42.3 (608)	35.3 (1692)	
**County**				< 0.001
Finnmark	70.2 (4426)	82.9 (1205)	66.4 (3221)	
Troms	17.1 (1079)	10.5 (153)	19.1 (926)	
Nordland/Trøndelag	12.7 (798)	6.6 (96)	14.5 (702)	
**Childhood Violence**				< 0.001
No	76.7 (4837)	68.6 (998)	79.2 (3839)	
Yes	23.3 (1466)	31.4 (456)	20.8 (1010)	
**Mental health problems**				0.002
No	86.6 (5210)	84.1 (1177)	87.4 (4033)	
Yes	13.4 (805)	15.9 (222)	12.6 (583)	

*Abbreviations* SD, standard deviation; p-value; Comparing Sami and non-Sami by Pearson’s chi-square test. Comparing means with two- sample t-test. Education level missing *n* = 72 (1.1%), mental health problems last 4 weeks: 10-item version of the Hopkins Symptoms Check list (HSCL-10) with a cut-off off ≥ 1.85, missing *n* = 178 (2.8%)

**Table 2:** Background characteristics among all men and by ethnicity. The SAMINOR 2 Questionnaire Survey and the Norwegian prescription database 2004–2019

	All men % *n* = 4993	Sami % *n* = 1104	Non-Sami % *n* = 3889	*P*
**Age (years)**, **mean (SD)**	49.7 (13.4)	49.9 (13.2)	49.6 (13.5)	0.415
**Educational level**, **% (n)**				0.184
Primary school	18.3 (903)	20.2 (221)	17.7 (682)	
Secondary school	31.1 (1534)	31.6 (345)	30.9 (1189)	
College/university 1–3 years	25.6 (1262)	24.0 (262)	26.0 (1000)	
College/university ≥ 4 years	25.1 (1238)	24.2 (264)	25.3 (974)	
**County**				< 0.001
Finnmark	66.6 (3325)	78.5 (867)	63.2 (2388)	
Troms	19.0 (950)	13.8 (152)	20.5 (773)	
Nordland/Trøndelag	14.4 (718)	7.7 (85)	16.3 (617)	
**Childhood Violence**				< 0.001
No	81.6 (4076)	72.0 (795)	84.4 (3281)	
Yes	18.4 (917)	28.0 (309)	15.6 (608)	
**Mental health problems**				0.005
No	91.4 (4385)	89.3 (951)	92.0 (3434)	
Yes	8.6 (412)	10.7 (114)	8.0 (298	

*Abbreviations* SD, standard deviation; p-value; Comparing Sami and non-Sami by Pearson’s chi-square test. Comparing means with two- sample t-test Education level missing *n* = 56 (1.1%), mental health problems last 4 weeks: 10-item version of the Hopkins Symptoms Check list (HSCL-10) with a cut-off off ≥ 1.85, missing *n* = 196 (3.9%)

**Fig. 1:**
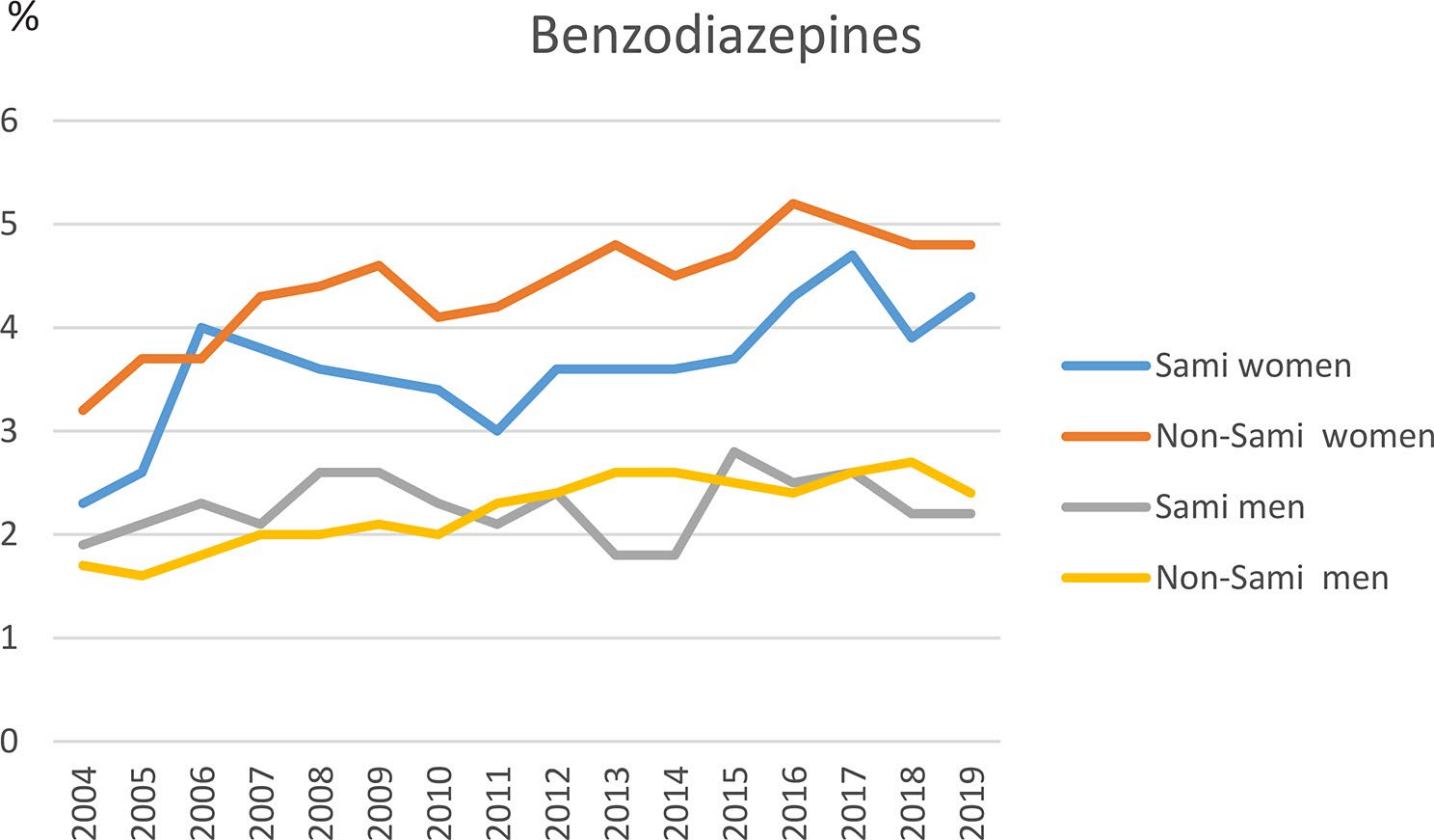
Prescriptions of benzodiazepines and antidepressant among all women and by ethnicity. The SAMINOR 2 Questionnaire Survey and the Norwegian Prescription Database 2004–2019

**Fig. 2:**
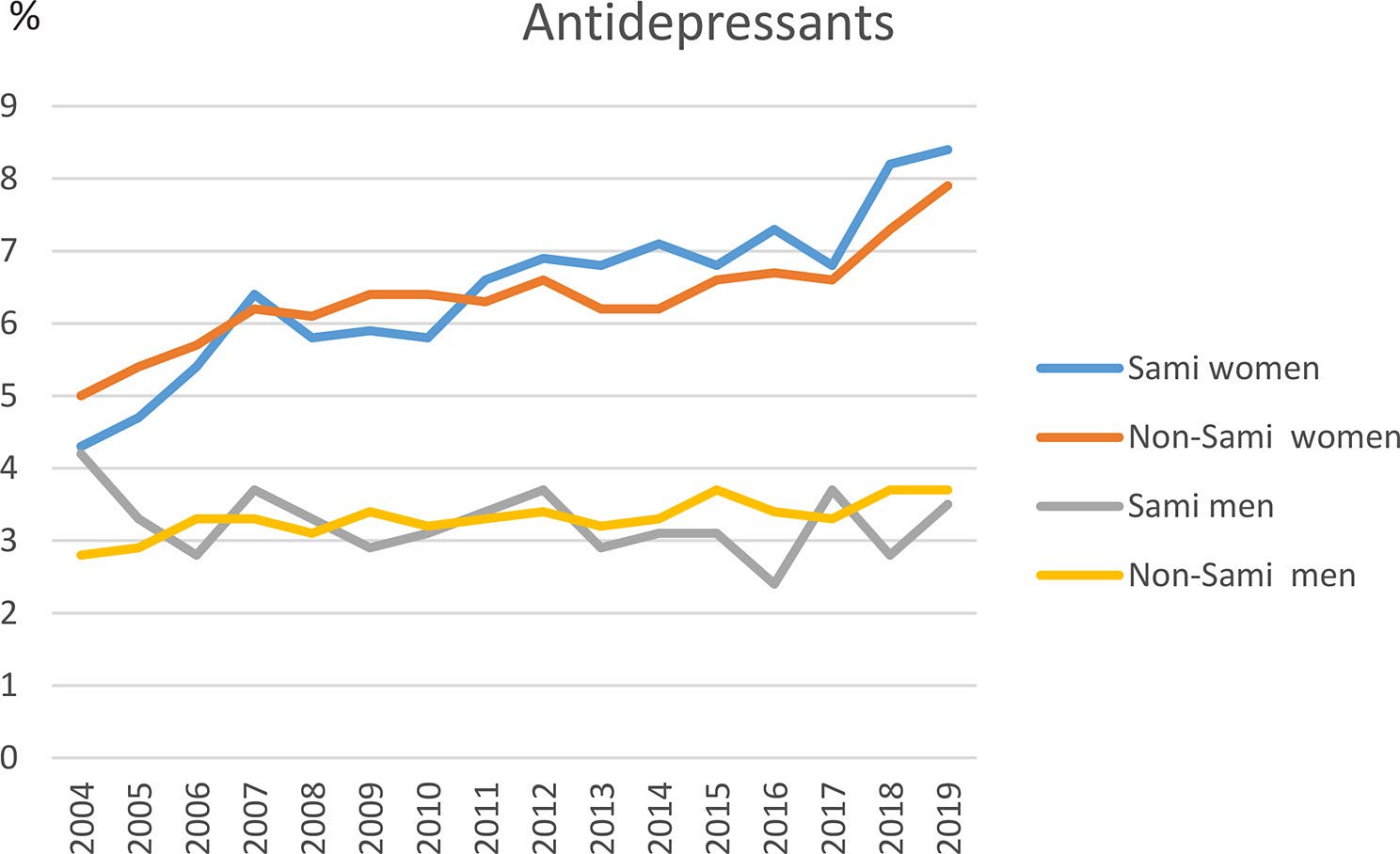
Prescriptions of benzodiazepines and antidepressant among all men and by ethnicity. The SAMINOR 2 Questionnaire Survey and the Norwegian Prescription Database 2004–2019

During the 16-year study period (2004–2019), 16.7% of all women filled at least one prescription for BDZ. Among Sami women the figure was 14.1% vs. 17.4% among non-Sami women (*p* = .003). Among all women, 23.6% filled at least one prescription for antidepressants, with no difference between ethnic groups (Figs. 1 and 2, supplemental file A). The usage of BDZ among men was 10.0% and the usage of antidepressants was 14.2%, with no difference between ethnic groups (Figs. 1 and 2, supplemental file B).

Studying each year separately, the prevalence of BDZ use among all women ranged between 3.0% and 5.0%; and the figures for Sami women ranged between 2.4% and 4.7%, vs. 3.2-5.0% among non-Sami women. A significantly lower proportion of Sami women compared to non-Sami women filled prescriptions for BDZ in 2005 (*p* = .035) and 2011 (*P* = .038), while no difference between ethnic groups was observed for other calendar years (Fig. 1, supplemental file A). The prevalence of antidepressants among all women each year ranged between 4.8% and 8.0%, with no differences between ethnic groups (Fig. 2, supplemental file A). Among all men the figures for BDZ each year ranged between 1.8% and 2.7%, and for antidepressants the figures were 3.0-3.7%, with no ethnic differences (Figs. 1 and 2, supplemental file B).

Investigating each year separately and in total, a significantly higher proportion of women than men filled prescriptions for BDZ and antidepressants, respectively (*p* ≤ .001 in all tests; results not shown in tables).

Among all women, a significantly higher proportion of those exposed to CV filled a prescription for BDZ and antidepressants than those not exposed to CV (p = < 0.001, Table 3). This was the case both when studying each year separately and when studying the period 2004–2019 as a whole. Stratified analyses by ethnicity showed the same results (supplemental file C & D). The same pattern was observed among all men for both BDZ and antidepressants (Table 4), except for BDZ in year 2010 (*p* = .091). Stratified analyses by ethnicity showed the same results. However, among Sami men the results showed the same pattern, but did not reach a level of significance, probably due to a lack of power (supplemental file E & F).

**Table 3:** Prescriptions of antidepressant and benzodiazepines among women exposed and not exposed to childhood violence. The SAMINOR 2 Questionnaire Survey and the Norwegian prescription database 2004–2019

All women	Not exposed to CV	Exposed to CV	*p*
**Benzodiazepines**			
**Year**	***% (n)***	***% (n)***	
2004	2.6 (124)	4.6 (68)	< 0.001
2005	3.1 (149)	4.9 (72)	0.001
2006	3.2 (153)	6.2 (91)	< 0.001
2007	3.7 (171)	6.1 (99)	< 0.001
2008	3.6 (176)	6.3 (93)	< 0.001
2009	3.8 (186)	6.4 (94)	< 0.001
2010	3.4 (164)	6.3 (92)	< 0.001
2011	3.6 (174)	5.8 (85)	< 0.001
2012	3.9 (188)	6.5 (95)	< 0.001
2013	3.9 (187)	7.1 (104)	< 0.001
2014	3.8 (184)	6.2 (90)	< 0.001
2015	4.0 (192)	6.6 (96)	< 0.001
2016	4.3 (207)	7.2 (105)	< 0.001
2017	4.1 (196)	7.8 (113)	< 0.001
2018	3.9 (184)	6.7 (97)	< 0.001
2019	4.0 (190)	6.5 (94)	< 0.001
Total 2012–2019	15.0 (709)	22.3 (231)	< 0.001
**Antidepressants**			
**Year**	***% (n)***	***% (n)***	**p**
2004	3.7 (177)	8.7 (127)	< 0.001
2005	4.1 (197)	9.2 (135)	< 0.001
2006	4.4 (215)	9.4 (138)	< 0.001
2007	5.0 (240)	10.4 (152)	< 0.001
2008	4.6 (223)	10.6 (156)	< 0.001
2009	5.0 (240)	10.4 (150)	< 0.001
2010	4.8 (231)	10.1 (148)	< 0.001
2011	4.9 (239)	11.0 (161)	< 0.001
2012	5.3 (256)	11.3 (165)	< 0.001
2013	5.0 (241)	10.9 (160)	< 0.001
2014	4.8 (232)	11.6 (169)	< 0.001
2015	5.1 (245)	11.7 (170)	< 0.001
2016	5.2 (250)	12.2 (177)	< 0.001
2017	5.3 (251)	11.1 (161)	< 0.001
2018	6.0 (286)	12.3 (178)	< 0.001
2019	6.5 (308)	13.0 (188)	< 0.001
Total 2004–2019	20.1 (950)	35.2 (507)	< 0.001

*Abbrevation* CV; exposure to childhood violence, Comparing those exposed to CV with those not exposed by Pearson’s chi-square test

**Table 4:** Prescriptions of antidepressant and benzodiazepines among all men exposed and not exposed to childhood violence. The SAMINOR 2 Questionnaire Survey and the Norwegian prescription database 2004–2019

All men	Not exposed to CV	Exposed to CV	*p*
**Benzodiazepines**			
**Year**	***% (n)***	***% (n)***	
2004	1.6 (67)	2.9 (27)	0.009
2005	1.6 (64)	2.9 (27)	0.005
2006	1.8 (72)	3.5 (32)	< 0.001
2007	1.9 (76)	3.5 (32)	0.002
2008	1.8 (75)	4.0 (37)	< 0.001
2009	2.1 (87)	3.9 (36)	0.002
2010	2.0 (83)	2.9 (27)	0.091
2011	2.0 (81)	4.4 (40)	< 0.001
2012	2.3 (92)	4.1 (37)	0.002
2013	2.1 (86)	4.3 (39)	< 0.001
2014	2.3 (91)	4.1 (37)	0.002
2015	2.3 (94)	4.2 (38)	0.002
2016	2.1 (82)	4.7 (42)	< 0.001
2017	2.3 (92)	3.8 (34)	0.010
2018	2.2 (88)	3.5 (31)	0.026
2019	1.8 (69)	4.9 (43)	< 0.001
Total 2004–2019	9.0 (350)	14.8 (129)	< 0.001
**Antidepressants**			
**Year**	***% (n)***	***% (n)***	**p**
2004	2.7 (112)	4.7 (43)	0.002
2005	2.6 (105)	4.8 (44)	< 0.001
2006	2.9 (117)	4.6 (42)	0.008
2007	2.7 (112)	6.1 (56)	< 0.001
2008	2.6 (104)	5.6 (51)	< 0.001
2009	2.9 (117)	5.2 (48)	< 0.001
2010	2.7 (109)	5.6 (51)	< 0.001
2011	2.7 (111)	6.1 (56)	< 0.001
2012	2.9 (118)	6.1 (56)	< 0.001
2013	2.6 (104)	5.5 (50)	< 0.001
2014	2.8 (113)	5.3 (48)	< 0.001
2015	3.0 (122)	5.8 (52)	< 0.001
2016	2.7 (106)	5.4 (48)	< 0.001
2017	2.8 (112)	6.0 (53)	< 0.001
2018	3.0 (119)	5.6 (49)	< 0.001
2019	3.3 (127)	5.6 (49)	< 0.001
Total 2004–2019	12.9 (502)	20.0 (174)	< 0.001

*Abbrevation* CV; exposure to childhood violence, Comparing those exposed to CV with those not exposed by Pearson’s chi-square test

**Fig. 3:**
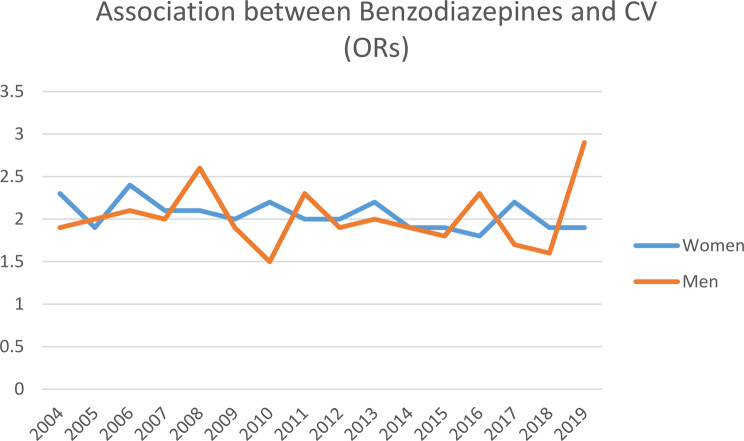
The effect of childhood violence on the use of benzodiazepines among women and men. The SAMINOR 2 Questionnaire Survey and the Norwegian Prescription Database 2004–2019. *Abbreviations* CV; exposure to childhood violence, OR; odds ratio, adjusted for age, reference group: not exposed to childhood violence

**Fig. 4:**
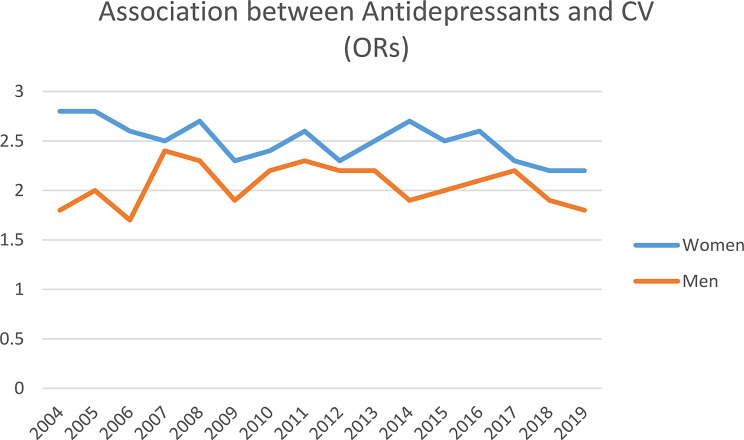
The effect of childhood violence on the use of antidepressant among women and men. The SAMINOR 2 Questionnaire Survey and the Norwegian Prescription Database 2004–2019. *Abbreviations* CV; exposure to childhood violence, OR; odds ratio, p; adjusted for age, reference group: not exposed to childhood violence

Among women, those exposed to CV had higher odds of receiving *one or more prescriptions* for BDZ and antidepressants separately for every year (2004–2019) and receiving BDZ and antidepressants in the years 2004–2019 combined (Figs. 3 and 4, supplemental file G). Adjusting for age in logistic regression analyses did not change the conclusion. Additional adjustment for educational level did not change the conclusion. Among men, the pattern was similar, with significant results for all calendar years except for BDZ in year 2010 (Figs. 3 and 4, supplemental file H). No significant interaction between CV and ethnicity was found.

Among all women, 7.7% (*n* = 483) were long-term users (≥ 2 years) of BDZ and 12.8% (*n* = 804) were long-term users of antidepressants, with no differences between ethnic groups (*p* = .199 and *p* = .500, respectively). A higher proportion of those exposed to CV were long-term users of BDZ (10.9%) and antidepressants (21.2%) than those not exposed to CV (6.7% and 10.2%, respectively, p = < 0.001). Among all men, 4.3% (*n* = 214) were long-term users of BDZ and 7.2% (*n* = 359) were long-term users of antidepressants, with no differences between ethnic groups (*p* = .824 and *p* = .729, respectively). A higher proportion of those exposed to CV were long-term users of BDZ (7.1%) and antidepressants (12.9%) compared to those not exposed to CV (3.7% and 5.9%, respectively, p = < 0.001 in both cases).

## Discussion

To our knowledge, this is the first study to examine filled prescriptions for BDZ and antidepressants among Sami compared to non-Sami men and women, and to investigate the effect of CV on the use of BDZ and antidepressants across these subpopulations.

During the 16-year study period (2004–2019), a lower proportion of Sami women filled prescriptions for BDZ compared to non-Sami women, while there was no significant difference between ethnic groups in filled prescriptions for antidepressants. Among men, we found no differences between ethnic groups in filled prescriptions for either BDZ or antidepressants. A higher proportion of men and women exposed to CV received at least one prescription for BDZ and antidepressants compared to those not exposed to CV, with no differences between ethnic groups.

National statistics on drug consumption from 2004 to 2019 show higher prevalences of BDZ and antidepressant use than in our study. The general adult prevalence of BDZ and antidepressants use among women were respectively 5.3-7.6% and 8.1-10.6% in the period 2004–2019 (https://www.reseptregisteret.no/Prevalens). The figures among men were 3.2–4.3% and 4.2-6.0% respectively. However, the majority of the respondents in our study were residents in Troms and Finnmark counties. Looking at the prevalence in Troms and Finnmark county, a lower prevalence for both BDZ and antidepressants are found for both women and men compared to the prevalence at a national level; for women the prevalence were 5.2-6.8% and 6.9–8.6% respectively. The prevalence for men were 3.2-3.9% and 3.7-4.9% respectively (https://www.reseptregisteret.no/Prevalens). These figures are closer to our findings. A study based on data from NorPD showed an increase in BDZ usage in the years 2004–2008/2010, after which it decreased towards 2020 [10]. Use of antidepressants was relatively stable in this period, but increased slightly in 2020 and 2021, possibly due to the corona pandemic [11]. The result from our study is pre-corona-pandemic. One reason for a lower prevalence in our study compared to the national figures, and also to Troms and Finnmark county, might be that those with severe mental health problems did not participate in surveys like SAMINOR 2. However, the aim of the study was not to report the prevalence in this population, but to investigate any ethic differences in use.

It has been argued that prescription of psychotropic medications has become the predominant response to mental health problems [20]. Due to a previous finding of a higher prevalence of mental health problems among Sami than non-Sami, we expected a higher prevalence of filled prescriptions for BDZ and antidepressants among Sami than non-Sami, yet our results did not support this hypothesis. The findings even showed a lower total filled prescription rate for BDZ among Sami women compared to non-Sami women. This is in line with other studies that have found that minority groups were less likely to get prescriptions for psychotropic medications [20, 21].

BDZ and antidepressants can have a range of side effects such as addiction, anxiety, sleep disturbance and dizziness [12–15, 35]. It is possible that Sami encounter barriers to treatment, as cultural differences between physicians and minority patients may impede communication and hence the prescription of BDZ and antidepressants. Sami might also have a lower tendency to mention symptoms of mental health problems to the physician. Norris et al. argue that low utilisation of medication in some groups may be part of a wider pattern of lack of access to, or less optimal interaction with, the health system [20]. Greater stigma among Sami towards mental health problems may also partially explain disparities in filled prescriptions for BDZ [36]. Sami might also be more likely than non-Sami to view mental health problems as an issue that should be treated with healing, rather than/or in combination with medication [37]. Studies have found that Sami more often use traditional medicine than non-Sami [38, 39], also when suffering from mental health problems [39]. Another possible explanation might be that Sami have more adequate coping mechanisms to handle anxiety (like stronger social support). The reason for this finding is unknown and motivates further research into the reason that, despite a higher prevalence of mental health problems among Sami, there was no difference between ethnic groups in filled prescriptions for BDZ among men, and no difference between ethnic groups in filled prescriptions for antidepressants among Sami than non-Sami, and a lower proportion of Sami women in filled prescriptions for BDZ than non-Sami women.

It is also worth mentioning that our findings reveal a high proportion of long-term users of BDZ and antidepressants, in contrast to the national guidelines that urge short-term use [35]. The positive effect of the use of antidepressants for longer than six months is disputed and scientific documentation of a positive outcome of long-term use is lacking [40]. Future studies should put greater emphasis on this group.

### The effect of childhood violence on filled prescriptions for BDZ and antidepressants

This study finds that, among all women and men, those with a history of childhood violence were twice as likely to receive prescriptions for BDZ and antidepressants than those without a history of childhood violence (Figs. 3 and 4, supplemental file G and H). This finding is not unexpected, considering the strong association between CV and mental health problems, and that both BDZ and antidepressants are used to treat mental health problems. The association between childhood violence and use of psychotropic medication (BZD and antidepressants) in adulthood is sparsely investigated [41], although Nicolaidis et al. found that those exposed to childhood trauma used more antidepressants than those not exposed [42]. Considering the high prevalence of CV in Norway and globally, public health interventions should focus on preventing CV, to reduce the personal and societal burden of both mental health problems and CV. Since the cause of mental health problems is multifactorial, this result signals the need for healthcare professionals to screen for CV among individuals struggling with mental health problems and using BZD and antidepressants. Eliciting a history of CV can help identify individuals who are at high risk of developing mental health problems. Cognitive therapy, which has been demonstrated as effective to manage the negative consequences of CV, could then be offered as a treatment [43, 44]. Also, it is probably important to further develop tools and/or training to support clinicians in screening and managing the negative consequences of CV among patients with anxiety and depression. A study of rape survivors found that the majority of those who received a prescription for a sedative and/or antidepressant did so without disclosing the assault [45].

Previous research has found that Sami men disclose violence to professionals less than non-Sami men [46] This could lead to higher rates of unresolved trauma and possibly higher rates of BDZ and anti-depressant prescription, although our results do not support this, as we found no evidence of Sami/non-Sami differences in filled prescriptions for BDZ and antidepressants among men.

The proportion of long-term users of both BDZ and antidepressants is higher among those exposed to CV than others and might imply a greater need for symptom relief in this group. Future studies should put greater emphasis on this group.

A major strength of our study is that NorPD provides accurate data on filled prescriptions for BDZ and antidepressants and allows for longitudinal analyses at the individual level, which strengthens the validity of the study. The questions used to assess violence were taken from NorAQ. A validation study among women and men showed that NorAQ had good validity and reliability [47]. However, the questions used in this study represent a modified version of NorAQ, and they have not been validated for the Sami population or for the non-Sami population in rural Northern Norway. Thus, differences in cultural and linguistic interpretation may have influenced the observed differences between the two groups. However, the questions were formulated rather broadly, which may reduce potential bias based on cultural differences.

One major limitation of this study is the low participation rate in SAMINOR 2, which most likely introduced selection bias. Therefore, estimates of CV and BDZ and antidepressant use must be interpreted with caution. The information concerning non-respondents is sparse, other than that the participation rate increased with age and female gender (36). Whether there are differences between ethnic groups in terms of participation rates cannot be determined, as ethnicity is not recorded in any official register in Norway. Misclassification of ethnicity might have introduced bias. Misclassification of non-Sami into the Sami group is unlikely. However, misclassification of Sami into the non-Sami group is possible. There is no consensus on how to define Sami ethnicity, and different researchers use different criteria. Hence, some of the respondents defined as non-Sami in our study might be defined as Sami according to another definition. A long history of national assimilation policy has led to a loss of language and culture among the Sami, and respondents may hide their Sami ethnicity due to stigma. In our study, participants had to consider themselves Sami in order to be categorised as Sami. This means that many of the subjects categorised as non-Sami have Sami ancestry. Nevertheless, we strongly believe that self-identification is the best and most ethical way of defining ethnic belonging, also supported by ILO Convention no. 169, article 1 (40). The possible misclassification of ethnicity is probably non-differential, and any possible association between ethnicity and the use of BDZ and antidepressants might be attenuated.

Recall bias for the exposure variable may have introduced bias, and also a general tendency to underreport tabooed topics such as CV, thereby diminishing the association with prescription of BDZ and antidepressants between those exposed to CV and those not exposed to CV.

The municipalities included in this study were selected due to their high number or proportion of Sami inhabitants, and they cover a large part of the main, traditional Sami settlement areas in Norway. The results cannot necessarily be generalised to the entire Sami and non-Sami populations of Norway.

Another limitation is that the information from NorPD only comprises filled prescriptions. It might be that some respondents received a prescription but never picked it up from the pharmacy. This might have influenced the finding but are considered as of minor importance for the results.

## Conclusion

This study demonstrates that a lower proportion of Sami women filled prescriptions for BDZ than non-Sami women, while no differences in antidepressant usage were found. Future studies should investigate the reasons for this difference between ethnic groups. There were no ethnic-group differences between Sami and the non-Sami men, indicating similar use of BDZ and antidepressants among Sami and non-Sami men in the core Sami areas of Norway. Sami and non-Sami victims of childhood violence received prescriptions for BDZ and antidepressants more frequently than others, which highlights the importance of preventing CV and identifying a history of CV when treating adults with mental health problems.

## Electronic supplementary material

Below is the link to the electronic supplementary material.

Supplementary Material 1

## Data Availability

The dataset during the current study was not publicly available due to the strict privacy policy by REK but is available from the corresponding author on reasonable request.
