# Supplementary material for: Prescription of benzodiazepines and antidepressants among Sami and non-Sami — How childhood violence shapes prescription patterns: the SAMINOR 2 questionnaire survey and the Norwegian prescription database
Source: BMC Public Health. 2024 Nov 7;24:3084. doi: 10.1186/s12889-024-20570-1 (PMC11546297; doi:10.1186/s12889-024-20570-1)
Supplement: Supplementary file 1 — Supplementary Material 1 [file 12889_2024_20570_MOESM1_ESM.docx]

# Prescription of Benzodiazepines and Antidepressants among Sami and non-Sami — How Childhood Violence Shapes Prescription Patterns: the SAMINOR 2 Questionnaire Survey and the Norwegian Prescription Database

**Supplemental file A. Prescriptions of benzodiazepines and antidepressant among all women and by ethnicity. The SAMINOR 2 Questionnaire Survey and the Norwegian Prescription Database 2004-2019.**

| **Benzodiazepines** |  | **All women** | **Sami** |  | **Non-Sami** |  |  |
| --- | --- | --- | --- | --- | --- | --- | --- |
| **Year** | **n=** | **% (n)** | **% (n)** | **n=** | **% (n)** | **n=** | **p** |
| **2004** | 6303 | 3.0 (192) | 2.4 (35) | 1454 | 3.2 (157) | 4849 | .106 |
| **2005** | 6303 | 3.5 (221) | 2.6 (38) | 1454 | 3.8 (183) | 4849 | .035 |
| **2006** | 6303 | 3.9 (244) | 4.0 (58) | 1454 | 3.8 (186) | 4849 | .791 |
| **2007** | 6303 | 4.3 (270) | 3.9 (57) | 1454 | 4.4 (213) | 4849 | .435 |
| **2008** | 6303 | 4.3 (269) | 3.7 (54) | 1454 | 4.4 (215) | 4849 | .234 |
| **2009** | 6303 | 4.4 (280) | 3.6 (53) | 1454 | 4.7 (227) | 4849 | .093 |
| **2010** | 6303 | 4.1 (256) | 3.4 (50) | 1454 | 4.2 (206) | 4849 | .170 |
| **2011** | 6303 | 4.1 (259) | 3.2 (46) | 1454 | 4.4 (213) | 4849 | .038 |
| **2012** | 6297 | 4.5 (283) | 3.9 (57) | 1453 | 4.7 (226) | 4844 | .231 |
| **2013** | 6286 | 4.6 (291) | 3.9 (57) | 1450 | 4.8 (234) | 4836 | .149 |
| **2014** | 6272 | 4.4 (274) | 3.7 (53) | 1444 | 4.6 (221) | 4828 | .139 |
| **2015** | 6257 | 4.6 (288) | 3.8 (55) | 1442 | 4.8 (233) | 4815 | .103 |
| **2016** | 6238 | 5.0 (312) | 4.3 (62) | 1439 | 5.2 (250) | 4799 | .169 |
| **2017** | 6217 | 5.0 (309) | 4.7 (68) | 1434 | 5.0 (241) | 4783 | .650 |
| **2018** | 6200 | 4.5 (281) | 3.9 (56) | 1434 | 4.7 (225) | 4766 | .193 |
| **2019** | 6178 | 4.6 (284) | 4.2 (60) | 1432 | 4.7 (224) | 4746 | .401 |
| **Total 2004-2019** | 6178 | 16.7 (1030) | 14.1 (202) | 1432 | 17.4 (828) | 4746 | .003 |
| **Antidepressants** |  | **All women** | **Sami** |  | **Non-Sami** |  | **p** |
| **Year** | **n=** | **% (n)** | **% (n)** | **n=** | **% (n)** | **n=** |  |
| **2004** | 6303 | 4.8 (304) | 4.3 (62) | 1454 | 5.0 (242) | 4849 | .257 |
| **2005** | 6303 | 5.3 (332) | 4.7 (69) | 1454 | 5.4 (263) | 4849 | .310 |
| **2006** | 6303 | 5.6 (353) | 5.4 (79) | 1454 | 5.7 (274) | 4849 | .752 |
| **2007** | 6303 | 6.2 (392) | 6.4 (93) | 1454 | 6.2 (299) | 4849 | .750 |
| **2008** | 6303 | 6.0 (379) | 5.8 (85) | 1454 | 6.1 (294) | 4849 | .760 |
| **2009** | 6303 | 6.2 (390) | 5.9 (84) | 1454 | 6.4 (306) | 4849 | .460 |
| **2010** | 6303 | 6.0 (393) | 5.8 (85) | 1454 | 6.4 (308) | 4849 | .484 |
| **2011** | 6303 | 6.3 (400) | 6.6 (96) | 1454 | 6.3 (304) | 4849 | .648 |
| **2012** | 6297 | 6.7 (421) | 6.9 (100) | 1453 | 6.6 (321) | 4844 | .732 |
| **2013** | 6286 | 6.4 (401) | 6.8 (99) | 1450 | 6.2 (302) | 4836 | .426 |
| **2014** | 6272 | 6.4 (401) | 7.1 (102) | 1444 | 6.2 (299) | 4828 | .235 |
| **2015** | 6257 | 6.6 (415) | 6.8 (98) | 1442 | 6.6 (317) | 4815 | .776 |
| **2016** | 6238 | 6.8 (427) | 7.3 (105) | 1439 | 6.7 (322) | 4799 | .439 |
| **2017** | 6217 | 6.6 (412) | 6.8 (97) | 1434 | 6.6 (315) | 4783 | .812 |
| **2018** | 6200 | 7.5 (464) | 8.2 (118) | 1434 | 7.3 (346) | 4766 | .221 |
| **2019** | 6178 | 8.0 (496) | 8.4 (121) | 1432 | 7.9 (375) | 4746 | .503 |
| **Total 2004-2019** | 6178 | 23.6 (1457) | 24.6 (352) | 1432 | 23.3 (1105) | 4746 | .310 |

**Supplemental file B. Prescriptions of benzodiazepines and antidepressant among all men and by ethnicity. The SAMINOR 2 Questionnaire Survey and the Norwegian Prescription Database 2004-2019.**

| **Benzodiazepines** |  | **All men** | **Sami** |  | **Non-Sami** |  |  |
| --- | --- | --- | --- | --- | --- | --- | --- |
| **Year** | **n=** | **% (n)** | **% (n)** | **n=** | **% (n)** | **n=** | **p** |
| **2004** | 4993 | 1.9 (94) | 1.9 (21) | 1104 | 1.9 (73) | 3878 | .957 |
| **2005** | 4993 | 1.8 (91) | 2.1 (23) | 1104 | 1.7 (68) | 3878 | .463 |
| **2006** | 4993 | 2.1 (104) | 2.4 (26) | 1104 | 2.0 (78) | 3878 | .473 |
| **2007** | 4993 | 2.2 (108) | 2.1 (23) | 1104 | 2.2 (85) | 3878 | .837 |
| **2008** | 4993 | 2.2 (112) | 2.6 (29) | 1104 | 2.1 (83) | 3878 | .329 |
| **2009** | 4993 | 2.5 (123) | 2.6 (29) | 1104 | 2.4 (94) | 3878 | .692 |
| **2010** | 4993 | 2.2 (110) | 2.4 (26) | 1104 | 2.2 (84) | 3878 | .697 |
| **2011** | 4993 | 2.4 (121) | 2.2 (24) | 1104 | 2.5 (97) | 3878 | .541 |
| **2012** | 4976 | 2.6 (129) | 2.7 (30) | 1098 | 2.6 (99) | 3878 | .741 |
| **2013** | 4956 | 2.5 (125) | 1.8 (20) | 1094 | 2.7 (105) | 3862 | .097 |
| **2014** | 4930 | 2.6 (128) | 1.9 (21) | 1088 | 2.8 (107) | 3842 | .118 |
| **2015** | 4898 | 2.7 (132) | 2.9 (31) | 1080 | 2.6 (101) | 3818 | .687 |
| **2016** | 4866 | 2.5 (124) | 2.5 (27) | 1071 | 2.6 (97) | 3795 | .949 |
| **2017** | 4846 | 2.6 (126) | 2.6 (28) | 1068 | 2.6 (98) | 3778 | .960 |
| **2018** | 4810 | 2.5 (119) | 2.1 (22) | 1057 | 2.6 (97) | 3753 | .352 |
| **2019** | 4773 | 2.3 (112) | 2.2 (23) | 1049 | 2.4 (89) | 3724 | .709 |
| **Total 2004-2019** | 4773 | 10.0 (479) | 9.6 (101) | 1049 | 10.2 (378) | 3724 | .619 |
| **Antidepressants** |  | **All men** | **Sami** |  | **Non-Sami** |  |  |
| **Year** | **n=** | **% (n)** | **% (n)** | **n=** | **% (n)** | **n=** | **p** |
| **2004** | 4993 | 3.1 (155) | 4.2 (46) | 1104 | 2.8 (109) | 3878 | .021 |
| **2005** | 4993 | 3.0 (149) | 3.3 (36) | 1104 | 2.9 (113) | 3878 | .540 |
| **2006** | 4993 | 3.2 (159) | 2.8 (31) | 1104 | 3.3 (128) | 3878 | .420 |
| **2007** | 4993 | 3.4 (168) | 3.7 (41) | 1104 | 3.3 (127) | 3878 | .466 |
| **2008** | 4993 | 3.1 (155) | 3.3 (36) | 1104 | 3.1 (119) | 3878 | .734 |
| **2009** | 4993 | 3.3 (165) | 2.9 (32) | 1104 | 3.4 (133) | 3878 | .392 |
| **2010** | 4993 | 3.2 (160) | 3.1 (34) | 1104 | 3.2 (126) | 3878 | .790 |
| **2011** | 4993 | 3.3 (167) | 3.4 (37) | 1104 | 3.3 (130) | 3878 | .989 |
| **2012** | 4976 | 3.5 (174) | 3.7 (41) | 1098 | 3.4 (133) | 3878 | .628 |
| **2013** | 4956 | 3.1 (154) | 2.9 (32) | 1094 | 3.2 (122) | 3862 | .694 |
| **2014** | 4930 | 3.3 (161) | 3.1 (34) | 1088 | 3.3 (127) | 3842 | .767 |
| **2015** | 4898 | 3.6 (174) | 3.1 (33) | 1080 | 3.7 (141) | 3818 | .318 |
| **2016** | 4866 | 3.2 (154) | 2.4 (26) | 1071 | 3.4 (128) | 3795 | .119 |
| **2017** | 4846 | 3.4 (165) | 3.7 (39) | 1068 | 3.3 (126) | 3778 | .614 |
| **2018** | 4810 | 3.5 (168) | 2.8 (30) | 1057 | 3.7 (138) | 3753 | .189 |
| **2019** | 4773 | 3.7 (176) | 3.5 (37) | 1049 | 3.7 (139) | 3724 | .755 |
| **Total 2004-2019** | 4773 | 14.2 (676) | 14.4 (151) | 1049 | 14.1 (525) | 3724 | .808 |

**Supplemental file C. Prescriptions of benzodiazepines among all women exposed and not exposed to childhood violence and by ethnicity. The SAMINOR 2 Questionnaire Survey and the Norwegian Prescription Database 2004-2019.**

| **Benzodiazepines** | **All women** |  | **Sami** |  | **Non-Sami** |  |
| --- | --- | --- | --- | --- | --- | --- |
| **Year** | **% (n)** | **p** | **% (n)** | **p** | **% (n)** | **p** |
| **2004** |  | <.001 |  | .003 |  | .001 |
| **No** | 2.6 (124) |  | 1.6 (16) |  | 2.8 (108) |  |
| **Yes** | 4.6 (68) |  | 4.2 (19) |  | 4.9 (49) |  |
| **2005** |  | <.001 |  | .031 |  | .003 |
| **No** | 3.1 (149) |  | 2.0 (20) |  | 3.4 (129) |  |
| **Yes** | 4.9 (72) |  | 3.9 (18) |  | 5.3 (54) |  |
| **2006** |  | <.001 |  | .002 |  | <.001 |
| **No** | 3.2 (153) |  | 2.9 (29) |  | 3.2 (124) |  |
| **Yes** | 6.2 (91) |  | 6.4 (29) |  | 6.1 (62) |  |
| **2007** |  | <.001 |  | .074 |  | <.001 |
| **No** | 3.7 (177) |  | 3.3 (33) |  | 3.8 (144) |  |
| **Yes** | 6.3 (93) |  | 5.3 (24) |  | 6.8 (69) |  |
| **2008** |  | <.001 |  | .003 |  | <.001 |
| **No** | 3.6 (176) |  | 2.7 (27) |  | 3.9 (149) |  |
| **Yes** | 6.3 (93) |  | 5.9 (27) |  | 6.5 (66) |  |
| **2009** |  | <.001 |  | .054 |  | <.001 |
| **No** | 3.8 (186) |  | 3.0 (30) |  | 4.1 (156) |  |
| **Yes** | 6.4 (94) |  | 5.0 (23) |  | 7.0 (71) |  |
| **2010** |  | <.001 |  | .099 |  | <.001 |
| **No** | 3.4 (164) |  | 2.9 (29) |  | 3.5 (135) |  |
| **Yes** | 6.3 (92) |  | 4.6 (21) |  | 7.0 (71) |  |
| **2011** |  | <.001 |  | .034 |  | <.001 |
| **No** | 3.6 (174) |  | 2.5 (25) |  | 3.9 (149) |  |
| **Yes** | 5.8 (85) |  | 4.6 (21) |  | 6.3 (64) |  |
| **2012** |  | <.001 |  | .019 |  | <.001 |
| **No** | 3.8 (179) |  | 2.8 (28) |  | 4.0 (151) |  |
| **Yes** | 6.2 (90) |  | 5.3 (24) |  | 6.6 (66) |  |
| **2013** |  | <.001 |  | <.001 |  | <.001 |
| **No** | 3.8 (179) |  | 2.3 (23) |  | 4.1 (156) |  |
| **Yes** | 7.0 (101) |  | 6.4 (29) |  | 7.2 (72) |  |
| **2014** |  | <.001 |  | .014 |  | .002 |
| **No** | 3.7 (178) |  | 2.7 (27) |  | 4.0 (151) |  |
| **Yes** | 6.0 (87) |  | 5.3 (24) |  | 6.3 (63) |  |
| **2015** |  | <.001 |  | .012 |  | <.001 |
| **No** | 3.9 (185) |  | 2.8 (28) |  | 4.1 (157) |  |
| **Yes** | 6.6 (95) |  | 5.6 (25) |  | 7.0 (70) |  |
| **2016** |  | <.001 |  | .027 |  | <.001 |
| **No** | 4.3 (205) |  | 3.5 (34) |  | 4.5 (171) |  |
| **Yes** | 7.1 (103) |  | 6.0 (27) |  | 7.6 (76) |  |
| **2017** |  | <.001 |  | .040 |  | <.001 |
| **No** | 4.1 (196) |  | 4.0 (39) |  | 4.1 (157) |  |
| **Yes** | 7.8 (113) |  | 6.4 (29) |  | 8.4 (84) |  |
| **2018** |  | <.001 |  | .013 |  | <.001 |
| **No** | 4.0 (189) |  | 3.0 (30) |  | 4.2 (159) |  |
| **Yes** | 6.8 (98) |  | 5.8 (26) |  | 7.2 (72) |  |
| **2019** |  | <.001 |  | .321 |  | <.001 |
| **No** | 4.1 (194) |  | 4.0 (39) |  | 4.1 (155) |  |
| **Yes** | 6.8 (98) |  | 5.1 (23) |  | 7.5 (75) |  |
| **Total 2012-2019** |  | <.001 |  | .002 |  | <.001 |
| **No** | 10.5 (500) |  | 8.7 (86) |  | 10.9 (414) |  |
| **yes** | 16.0 (232) |  | 14.0 (63) |  | 16.9 (169) |  |

**Supplemental file D. Prescriptions of antidepressant among all women exposed and not exposed to childhood violence by ethnicity. The SAMINOR 2 Questionnaire Survey and the Norwegian Prescription Database 2004-2019.**

| **Antidepressant** | **All women** |  | **Sami** |  | **Non-Sami** |  |
| --- | --- | --- | --- | --- | --- | --- |
| **Year** | **% (n)** | **p** | **% (n)** |  | **% (n)** | **p** |
| **2004** |  | <.001 |  | <.001 |  | <.001 |
| **No** | 3.7 (177) |  | 2.5 (25) |  | 4.0 (152) |  |
| **Yes** | 8.7 (127) |  | 8.1 (37) |  | 8.9 (90) |  |
| **2005** |  | <.001 |  | <.001 |  | <.001 |
| **No** | 4.1 (197) |  | 3.0 (30) |  | 4.4 (167) |  |
| **Yes** | 9.2 (135) |  | 8.6 (39) |  | 9.5 (96) |  |
| **2006** |  | <.001 |  | <.001 |  | <.001 |
| **No** | 4.4 (215) |  | 4.1 (41) |  | 4.5 (174) |  |
| **Yes** | 9.4 (138) |  | 8.3 (38) |  | 9.9 (100) |  |
| **2007** |  | <.001 |  | <.001 |  | <.001 |
| **No** | 5.0 (240) |  | 4.8 (48) |  | 5.0 (192) |  |
| **Yes** | 10.4 (152) |  | 9.9 (45) |  | 10.6 (107) |  |
| **2008** |  | <.001 |  | .006 |  | <.001 |
| **No** | 4.6 (223) |  | 4.7 (47) |  | 4.6 (176) |  |
| **Yes** | 10.6 (156) |  | 8.3 (38) |  | 11.7 (118) |  |
| **2009** |  | <.001 |  | .006 |  | <.001 |
| **No** | 5.0 (243) |  | 4.7 (47) |  | 5.1 (196) |  |
| **Yes** | 10.2 (150) |  | 8.3 (38) |  | 11.1 (112) |  |
| **2010** |  | <.001 |  | <.001 |  | <.001 |
| **No** | 4.8 (231) |  | 4.6 (46) |  | 4.8 (185) |  |
| **Yes** | 10.1 (148) |  | 10.1 (46) |  | 10.1 (102) |  |
| **2011** |  | <.001 |  | <.001 |  | <.001 |
| **No** | 4.9 (239) |  | 5.0 (50) |  | 4.9 (189) |  |
| **Yes** | 11.0 (161) |  | 10.1 (46) |  | 11.4 (115) |  |
| **2012** |  | <.001 |  | <.001 |  | <.001 |
| **No** | 5.3 (251) |  | 4.8 (47) |  | 5.4 (204) |  |
| **Yes** | 11.1 (161) |  | 11.1 (50) |  | 11.1 (111) |  |
| **2013** |  | <.001 |  | <.001 |  | <.001 |
| **No** | 5.0 (238) |  | 4.9 (48) |  | 5.0 (190) |  |
| **Yes** | 10.9 (158) |  | 11.1 (50) |  | 10.8 (108) |  |
| **2014** |  | <.001 |  | <.001 |  | <.001 |
| **No** | 4.8 (228) |  | 4.9 (48) |  | 4.8 (180) |  |
| **Yes** | 11.5 (167) |  | 11.6 (52) |  | 11.5 (115) |  |
| **2015** |  | <.001 |  | <.001 |  | <.001 |
| **No** | 5.1 (241) |  | 4.5 (44) |  | 5.2 (197) |  |
| **Yes** | 11.7 (169) |  | 11.8 (53) |  | 11.6 (116) |  |
| **2016** |  | <.001 |  | <.001 |  | <.001 |
| **No** | 5.2 (250) |  | 4.8 (47) |  | 5.4 (203) |  |
| **Yes** | 12.2 (177) |  | 12.9 (58) |  | 11.9 (119) |  |
| **2017** |  | <.001 |  | <.001 |  | <.001 |
| **No** | 5.3 (251) |  | 4.8 (47) |  | 5.4 (204) |  |
| **Yes** | 11.1 (161) |  | 11.1 (50) |  | 11.1 (111) |  |
| **2018** |  | <.001 |  | <.001 |  | <.001 |
| **No** | 6.0 (287) |  | 6.1 (60) |  | 6.0 (227) |  |
| **Yes** | 12.3 (178) |  | 12.9 (58) |  | 12.0 (120) |  |
| **2019** |  | <.001 |  | <.001 |  | <.001 |
| **No** | 6.5 (311) |  | 6.6 (65) |  | 6.5 (246) |  |
| **Yes** | 13.1 (190) |  | 12.7 (57) |  | 13.3 (133) |  |
| **2020** |  | <.001 |  | <.001 |  | <.001 |
| **No** | 6.0 (287) |  | 6.0 (59) |  | 6.0 (228) |  |
| **Yes** | 12.9 (187) |  | 14.2 (64) |  | 12.3 (123) |  |
| **Total 2012-2019** |  | <.001 |  | <.001 |  | <.001 |
| **No** | 14.0 (667) |  | 13.3 (131) |  | 14.2 (536) |  |
| **Yes** | 26.0 (376) |  | 26.2 (118) |  | 25.9 (258) |  |

**Supplemental file E. Prescriptions of benzodiazepines among all men exposed and not exposed to childhood violence and by ethnicity. The SAMINOR 2 Questionnaire Survey and the Norwegian Prescription Database 2004-2019.**

| **Benzodiazepines** | **All men** |  | **Sami** | **p** | **Non-Sami** |  |
| --- | --- | --- | --- | --- | --- | --- |
| **Year** | **% (n)** | **p** | **% (n)** |  | **% (n)** | **p** |
| **2004** |  | .009 |  | .125 |  | .032 |
| **No** | 1.6 (67) |  | 1.5 (12) |  | 1.7 (55) |  |
| **Yes** | 2.9 (27) |  | 2.9 (9) |  | 3.0 (18) |  |
| **2005** |  | .005 |  | .032 |  | .071 |
| **No** | 1.6 (64) |  | 1.5 (12) |  | 1.6 (52) |  |
| **Yes** | 2.9 (27) |  | 3.6 (11) |  | 2.6 (16) |  |
| **2006** |  | <.001 |  | .003 |  | .067 |
| **No** | 1.8 (72) |  | 1.5 (12) |  | 1.8 (60) |  |
| **Yes** | 3.5 (32) |  | 4.5 (14) |  | 3.0 (18) |  |
| **2007** |  | <.001 |  | .837 |  | .002 |
| **No** | 1.9 (76) |  | 2.1 (17) |  | 1.8 (59) |  |
| **Yes** | 3.5 (32) |  | 1.9 (6) |  | 4.3 (26) |  |
| **2008** |  | <.001 |  | .041 |  | <.001 |
| **No** | 1.8 (75) |  | 2.0 (16) |  | 1.8 (59) |  |
| **Yes** | 4.0 (37) |  | 4.2 (13) |  | 3.9 (24) |  |
| **2009** |  | .002 |  | .711 |  | <.001 |
| **No** | 2.1 (87) |  | 2.5 (20) |  | 2.0 (67) |  |
| **Yes** | 3.9 (36) |  | 2.9 (9) |  | 4.4 (27) |  |
| **2010** |  | .091 |  | .749 |  | .075 |
| **No** | 2.0 (83) |  | 2.3 (18) |  | 2.0 (65) |  |
| **Yes** | 2.9 (27) |  | 2.6 (8) |  | 3.1 (19) |  |
| **2011** |  | <.001 |  | .294 |  | <.001 |
| **No** | 2.0 (81) |  | 1.9 (15) |  | 2.0 (66) |  |
| **Yes** | 4.4 (40) |  | 2.9 (9) |  | 5.1 (31) |  |
| **2012** |  | <.001 |  | .245 |  | <.001 |
| **No** | 2.8 (112) |  | 3.2 (25) |  | 2.7 (87) |  |
| **Yes** | 6.0 (53) |  | 4.7 (14) |  | 6.6 (39) |  |
| **2013** |  | <.001 |  | .073 |  | <.001 |
| **No** | 2.5 (99) |  | 2.3 (18) |  | 2.5 881) |  |
| **Yes** | 5.4 (48) |  | 4.4 (13) |  | 6.0 (35) |  |
| **2014** |  | <.001 |  | .464 |  | <.001 |
| **No** | 2.7 (108) |  | 2.8 (22) |  | 2.7 (86) |  |
| **Yes** | 5.2 (46) |  | 3.7 (11) |  | 6.0 (35) |  |
| **2015** |  | <.001 |  | .128 |  | <.001 |
| **No** | 3.0 (119) |  | 2.6 (20) |  | 3.1 (99) |  |
| **Yes** | 5.8 (51) |  | 4.4 (13) |  | 6.5 (38) |  |
| **2016** |  | <.001 |  | .092 |  | <.001 |
| **No** | 2.7 (105) |  | 1.9 (15) |  | 2.8 (90) |  |
| **Yes** | 5.3 (53) |  | 3.7 (11) |  | 6.1 (36) |  |
| **2017** |  | <.001 |  | .245 |  | <.001 |
| **No** | 2.8 (112) |  | 3.2 (25) |  | 2.7 (87) |  |
| **Yes** | 6.0 (53) |  | 4.7 (14) |  | 6.6 (39) |  |
| **2018** |  | <.001 |  | .019 |  | .002 |
| **No** | 3.1 (121) |  | 2.1 (16) |  | 3.3 (105) |  |
| **Yes** | 5.5 (49) |  | 4.7 (14) |  | 6.0 (35) |  |
| **2019** |  | <.001 |  | .425 |  | <.001 |
| **No** | 3.3 (129) |  | 3.4 (26) |  | 3.2 (103) |  |
| **Yes** | 5.7 (50) |  | 4.4 (13) |  | 6.3 (37) |  |
| **Total 2012-2019** |  | <.001 |  | .063 |  | <.001 |
| **No** | 8.2 (324) |  | 8.2 (63) |  | 8.2 (261) |  |
| **Yes** | 13.2 (117) |  | 11.8 (35) |  | 13.9 (82) |  |

**Supplemental file F. Prescriptions of antidepressant among all men, and Sami and non-Sami men exposed and not exposed to childhood violence by ethnicity. The SAMINOR 2 Questionnaire Survey and the Norwegian Prescription Database 2004-2019.**

| **Antidepressants** | **All men** |  | **Sami** |  | **Non-Sami** |  |
| --- | --- | --- | --- | --- | --- | --- |
| **Year** |  | **p** |  | **p** |  | **p** |
| **2004** |  | .002 |  | .294 |  | .008 |
| **No** | 2.7 (112) |  | 3.8 (30) |  | 2.5 (82) |  |
| **Yes** | 4.7 (43) |  | 5.2 (16) |  | 4.4 (27) |  |
| **2005** |  | <.001 |  | .063 |  | .003 |
| **No** | 2.6 (105) |  | 2.6 821) |  | 2.6 (84) |  |
| **Yes** | 4.8 (44) |  | 4.9 (15) |  | 4.8 (29) |  |
| **2006** |  | .008 |  | .031 |  | .048 |
| **No** | 2.9 (117) |  | 2.1 (17) |  | 3.0 (100) |  |
| **Yes** | 4.6 (42) |  | 4.5 (14) |  | 4.6 (28) |  |
| **2007** |  | <.001 |  | .008 |  | <.001 |
| **No** | 2.7 (112) |  | 2.8 (22) |  | 2.7 (90) |  |
| **Yes** | 6.1 (56) |  | 6.1 (19) |  | 6.1 (37) |  |
| **2008** |  | <.001 |  | .003 |  | <.001 |
| **No** | 2.6 (104) |  | 2.3 (18) |  | 2.6 (86) |  |
| **Yes** | 5.6 (51) |  | 5.8 (18) |  | 5.4 (339 |  |
| **2009** |  | <.001 |  | .044 |  | .001 |
| **No** | 2.9 (117) |  | 2.3 (18) |  | 3.0 (99) |  |
| **Yes** | 5.2 (48) |  | 4.5 (14) |  | 5.6 (34) |  |
| **2010** |  | <.001 |  | .012 |  | <.001 |
| **No** | 2.7 (109) |  | 2.3 (18) |  | 2.8 (91) |  |
| **Yes** | 5.6 (51) |  | 5.2 (16) |  | 5.8 (35) |  |
| **2011** |  | <.001 |  | .036 |  | <.001 |
| **No** | 2.7 (111) |  | 2.6 (21) |  | 2.7 (90) |  |
| **Yes** | 6.1 (56) |  | 5.2 (16) |  | 6.6 (40) |  |
| **2012** |  | <.001 |  | .245 |  | <.001 |
| **No** | 2.8 (112) |  | 3.2 (25) |  | 2.7 (87) |  |
| **Yes** | 6.0 (53) |  | 4.7 (14) |  | 6.6 (39) |  |
| **2013** |  | <.001 |  | .073 |  | <.001 |
| **No** | 2.5 (99) |  | 2.3 (18) |  | 2.5 881) |  |
| **Yes** | 5.4 (48) |  | 4.4 (13) |  | 6.0 (35) |  |
| **2014** |  | <.001 |  | .464 |  | <.001 |
| **No** | 2.7 (108) |  | 2.8 (22) |  | 2.7 (86) |  |
| **Yes** | 5.2 (46) |  | 3.7 (11) |  | 6.0 (35) |  |
| **2015** |  | <.001 |  | .128 |  | <.001 |
| **No** | 3.0 (119) |  | 2.6 (20) |  | 3.1 (99) |  |
| **Yes** | 5.8 (51) |  | 4.4 (13) |  | 6.5 (38) |  |
| **2016** |  | <.001 |  | .092 |  | <.001 |
| **No** | 2.7 (105) |  | 1.9 (15) |  | 2.8 (90) |  |
| **Yes** | 5.3 (53) |  | 3.7 (11) |  | 6.1 (36) |  |
| **2017** |  | <.001 |  | .245 |  | <.001 |
| **No** | 2.8 (112) |  | 3.2 (25) |  | 2.7 (87) |  |
| **Yes** | 6.0 (53) |  | 4.7 (14) |  | 6.6 (39) |  |
| **2018** |  | <.001 |  | .019 |  | .002 |
| **No** | 3.1 (121) |  | 2.1 (16) |  | 3.3 (105) |  |
| **Yes** | 5.5 (49) |  | 4.7 (14) |  | 6.0 (35) |  |
| **2019** |  | <.001 |  | .425 |  | <.001 |
| **No** | 3.3 (129) |  | 3.4 (26) |  | 3.2 (103) |  |
| **Yes** | 5.7 (50) |  | 4.4 (13) |  | 6.3 (37) |  |
| **2020** |  | <.001 |  | .205 |  | <.001 |
| **No** | 3.1 (123) |  | 2.8 (22) |  | 3.2 (101) |  |
| **Yes** | 5.7 (50) |  | 4.4 (13) |  | 6.3 (37) |  |
| **Total 2012-2019** |  | <.001 |  | .063 |  | <.001 |
| **No** | 8.2 (324) |  | 8.2 (63) |  | 8.2 (261) |  |
| **Yes** | 13.2 (117) |  | 11.8 (35) |  | 13.9 (82) |  |

**Supplemental file G. The effect of childhood violence on the use of benzodiazepines and antidepressant among women. The SAMINOR 2 Questionnaire Survey and the Norwegian Prescription Database 2004-2019.**

| **All women** | **BDZ** |  | **AD** |  |
| --- | --- | --- | --- | --- |
| **Year** | **OR^*^** | **p** | **OR^*^** | **p** |
| **2004** | 2.3 | <.001 | 2.8 | <.001 |
| **2005** | 1.9 | <.001 | 2.8 | <.001 |
| **2006** | 2.4 | <.001 | 2.6 | <.001 |
| **2007** | 2.1 | <.001 | 2.5 | <.001 |
| **2008** | 2.1 | <.001 | 2.7 | <.001 |
| **2009** | 2.0 | <.001 | 2.3 | <.001 |
| **2010** | 2.2 | <.001 | 2.4 | <.001 |
| **2011** | 2.0 | <.001 | 2.6 | <.001 |
| **2012** | 2.0 | <.001 | 2.3 | <.001 |
| **2013** | 2.2 | <.001 | 2.5 | <.001 |
| **2014** | 1.9 | <.001 | 2.7 | <.001 |
| **2015** | 1.9 | <.001 | 2.5 | <.001 |
| **2016** | 1.8 | <.001 | 2.6 | <.001 |
| **2017** | 2.2 | <.001 | 2.3 | <.001 |
| **2018** | 1.9 | <.001 | 2.2 | <.001 |
| **2019** | 1.9 | <.001 | 2.2 | <.001 |
| **Total 2004-2019** | 1.8 | <.001 | 2.2 | <.001 |

Abbreviations: BDZ; benzodiazepines, AD; antidepressants, OR; odds ratio, p; p-value*adjusted for age, reference group: not exposed to childhood violence.

**Supplemental file H. The effect of childhood violence on the use of benzodiazepines and antidepressant among men. The SAMINOR 2 Questionnaire Survey and the Norwegian Prescription Database 2004-2019.**

| **All men** | **BDZ** |  | **AD** |  |
| --- | --- | --- | --- | --- |
| **Year** | **OR^*^** | **p** | **OR^*^** | **p** |
| **2004** | 1.9 | .006 | 1.8 | .001 |
| **2005** | 2.0 | .004 | 2.0 | <.001 |
| **2006** | 2.1 | <.001 | 1.7 | .005 |
| **2007** | 2.0 | .002 | 2.4 | <.001 |
| **2008** | 2.3 | <.001 | 2.3 | <.001 |
| **2009** | 1.9 | .001 | 1.9 | <.001 |
| **2010** | 1.5 | .065 | 2.2 | <.001 |
| **2011** | 2.3 | <.001 | 2.3 | <.001 |
| **2012** | 1.9 | .002 | 2.2 | <.001 |
| **2013** | 2.0 | <.001 | 2.2 | <.001 |
| **2014** | 1.9 | <.001 | 1.9 | <.001 |
| **2015** | 1.8 | .002 | 2.0 | <.001 |
| **2016** | 2.3 | <.001 | 2.1 | <.001 |
| **2017** | 1.7 | .007 | 2.2 | <.001 |
| **2018** | 1.6 | .021 | 1.9 | <.001 |
| **2019** | 2.9 | <.001 | 1.8 | .001 |
| **Total 2012-2019** | 1.9 | <.001 | 1.7 | <.001 |
|  |  |  |  |  |

Abbreviations: BDZ; benzodiazepines, AD; antidepressants, OR; odds ratio, p; p-value*adjusted for age, reference group: not exposed to childhood violence.
